# Cathepsin a upregulation in glioma: A potential therapeutic target associated with immune infiltration

**DOI:** 10.5937/jomb0-35677

**Published:** 2022-10-15

**Authors:** Ming Zhang, Jun Huang, Yunfei Wang, Qingbin Nie, Xinye Zhang, Yufeng Yang, Gengsheng Mao

**Affiliations:** 1 The Third Medical Centre Chinese PLA (People's Liberation Army) General Hospital, Department of Neurosurgery, Beijing, China

**Keywords:** cathepsin A, glioma, prognostic biomarker, immune infiltration, katepsin A, gliom, prognostički biomarker, imunolška infiltracija

## Abstract

**Background:**

Glioma is the result of malignant transformation of glial cells in the white matter of the brain or spinal cord and accounts for approximately 80% of all intracranial malignancies. Cathepsin A (CTSA) is highly expressed in a variety of tumor tissues, but its role in glioma is poorly studied. This study analyses the relationship between CTSA, and glioma based on The Cancer Genome Atlas (TCGA).

**Methods:**

Data for glioma patients were collected from TCGA. The expression level of CTSA was compared between paired glioma tissues and normal tissues with Wilcoxon rank-sum test. In addition, the Wilcoxon ranksum test was also applied to analyze the relationship between clinicopathologic features and CTSA expression. Kaplan-Meier Plotter was applied to analyze OS, DSS and PFI. Immuno-infiltration analysis of BLCA was performed by single sample gene set enrichment analysis (ssGSEA) in the "GSVA" R package.

**Results:**

The CTSA was overexpressed in glioma tissues compared to normal tissues (P<0.001). The high expression of CTSA was significantly related to 1p/19q codeletion, IDH, WHO grade and histological type. Kaplan-Meier survival analysis showed that patients with glioma characterized with high expressed CTSA had a poorer OS (HR=2.16 P<0.001), DSS (HR=2.17 P<0.001) and PFI (HR=1.48 P<0.001) than patients with low CTSA expression. Moreover, High expressed CTSA was associated with immune cell infiltration.

**Conclusions:**

CTSA may serve as a candidate prognostic biomarker for determining prognosis associated with immune infiltration in glioma.

## Abstract (sr)

**Uvod:**

Gliom je rezultat maligne transformacije glijalnih ćelija u beloj materiji mozga ili kičmene moždine i čini približno 80% svih intrakranijalnih maligniteta. Katepsin A (CTSA) je visoko eksprimiran u različitim tumorskim tkivima, ali je njegova uloga u gliomu slabo pročavana. Ova studija analizira odnos između CTSA i glioma na osnovu The Cancer Genome Atlas (TCGA).

**Metode:**

Podaci za pacijente sa gliomom su prikupljeni od TCGA. Nivo ekspresije CTSA je upoređen između uparenih tkiva glioma i normalnih tkiva pomoću Wilcoxon testa rangsume. Pored toga, Wilcoxon rank-sum test je takođe primenjen za analizu odnosa između kliničkopatoloških karakteristika i ekspresije CTSA. Kaplan-Meier ploter je primenjen za analizu OS, DSS i PFI. Imuno-infiltraciona analiza BLCA izvršena je analizom obogaćivanja skupa gena jednog uzorka (ssGSEA) u paketu "GSVA" R.

**Rezultati:**

CTSA je bio prekomerno izražen u tkivima glioma u poređenju sa normalnim tkivima (P<0,001). Visoka ekspresija CTSA bila je značajno povezana sa 1p/19k kodelecijom, IDH, VHO stepenom i histološkim tipom. Kaplan-Meierova analiza preživljavanja je pokazala da su pacijenti sa gliomom okarakterisanim visoko izraženim CTSA imali lošiji OS (HR=2,16 P<0,001), DSS (HR=2,17 P<0,001) i PFI (HR=1,48 P<0,001) od pacijenata sa niska ekspresija CTSA. Štaviše, visoko izraženi CTSA je bio povezan sa infiltracijom imunih ćelija.

**Zaključak:**

CTSA može poslužiti kao kandidat za prognostički biomarker za određivanje prognoze povezane sa imunološkom infiltracijom u gliomu.

## Introduction

Glioma is the result of malignant transformation of glial cells in the white matter of the brain or spinal cord and accounts for approximately 80% of all intracranial malignancies [1]. In addition, the incidence of glioma is increasing year on year with an annual growth rate of 1-2% and a 5-year survival rate of only 10-20% [2]. The current treatment of glioma is mainly based on microsurgery, precise local radiotherapy, and chemotherapy, combined with preoperative three-dimensional reconstruction, intraoperative visualization navigation and postoperative gene targeting therapy [3]. The accurate diagnosis and prognosis evaluation technology in the early stage of glioma is important for the patients with glioma. Therefore, to study the occurrence and development mechanism of cellular carcinogenesis from a biological perspective, then discover more effective molecular diagnostic markers, prognostic indicators and therapeutic targets is one of the important topics.

Cathepsin is an important lysosomal proteolytic enzyme, which is mainly responsible for degrading intracellular or extracellular substrates [4]. It is reported that cathepsin expression is detected in a variety of tumors. In addition, cathepsin expression id related to tumor development and drug resistance [5]. For example, the over-expression of cathepsin can activate ErbB carcinogenic pathway [6], thereby promoting invasion and metastasis of breast cancer [7], pancreatic cancer [8], HCC [9] and colorectal cancer [10]. Moreover, cathepsin K is closely related to the progression of prostate cancer [11]. Cathepsin A (CTSA) is a serine member of cathepsin family, which can protect β-galactosidase and neuraminidase-1 from proteolysis in vivo [12]. Studies have confirmed that overexpression of CTSA is associated with a variety of tumors. However, the role of CTSA in glioma remains unclear.

In this study, we try to prove the correlation between CTSA and glioma, then to analyze the prognostic role of CTSA in glioma based on RNA sequencing (RNA-seq) data from TCGA. Furthermore, we analyzed CTSA expression levels in glioma and normal tissue and determined the correlation between CTSA expression and patient prognosis in terms of overall survival (OS). In addition, we performed prognostic and clinical correlation analyses to explore the potential diagnostic and prognostic value of CTSA. Enrichment analysis, molecular interaction network analysis and immune infiltration correlation analysis were also used to determine its biological sig-nificance. Taken together, our study suggests that CTSA is an important independent predictor of glioma.

## Materials and methods

### TIMER, GEPIA, UALCAN

TIMER (https://cistrome.shinyapps.io/timer/) is an interactive portal for the study of CTSA expression in various cancer types assessed by »Diff Exp«. cancer-pku.cn/index.html) is a web portal for gene expression analysis based on TCGA and GTEx data. This study analyzed CTSA expression through TCGAgliomatool for in-depth analysis of transcriptomic data from The Cancer Genome Atlas (TCGA) and MET500. This study investigated CTSA expression in glioma and the relationship between CTSA and various clinicopathological parameters (1p/19q codeletion, IDH, WHO and Histological type) using UAL-CAN.

### RNA-seq data source

UCSC XENA (https://xenabrowser.net/datapages/) RNAseq data in TPM format from TCGA and GTEx, harmonized by the Toil process (PMID: 28398314). RNAseq data in TPM (transcripts per million reads) format were log2-transformed for analysis and comparison. Data were extracted from TCGA for GBMLGG (glioma, 689 cases) and the corresponding normal tissue (1157 cases) in GTEx. Data were divided into high CTSA expression and low CTSA expression groups according to the median CTSA expression.

### Analysis of immune cell infiltration in ssGSEA

The immune infiltration analysis of BLCA was carried out through the single sample Gene Set Enrichment Analysis (ssGESA) in the »GSVA« R software package [13]. The infiltration levels of 24 immune cell types were quantified by gene expression profiles [14]. In addition, Spearman correlation analysis was carried out to explore the relationship between immune cell infiltration and CTSA.

### Statistical analysis

The R package (v.3.6.1) was used for all statistical analyses. Wilcoxon rank sum test and single gene logistic regression were carried out to analyze the relationship between CTSA and clinicopathological characteristics. Kaplan-Meier survival was applied detect the relationship between clinicopathological characteristics and OS, DSS and PFI in patients with glioma. P<0.05 was considered a statistically significant difference.

## Results

### Overexpression of CTSA in glioma tissue

We analyzed the expression of CTSA in the pancancer databases of TCGA and CTEx. The results revealed that CTSA was highly expressed in 15 tumor tissues, including HCC, BRCA, DLBC, GBM, KIRC, LGG, LIHC, OV, PAAD, PCPG, PRAD, SKCM, TGCT, THYM, and UCEC. However, CTSA was overexpressed in CESC, CHOL, COAD, ESCA, HNSC, KICH, LAML, LUAD, LUSC, READ, STAD, THCA, UCS were less expressed , the expression of CTSA was significantly higher in glioma tissues than in paraneoplastic tissues, which expressed that CSTA was upregulated in glioma tissues, implying that CSTA may play an important regulatory role in the progression of glioma.

**Figure 1:**
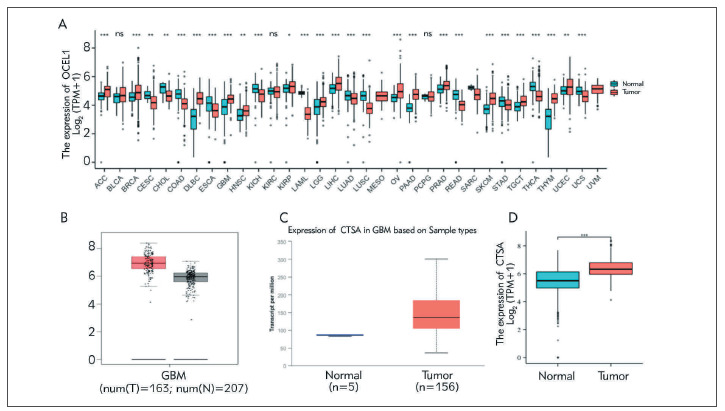
Expression of CTSA in glioma. (A) CTSA expression in different types of cancer was detected with TIMER database. (B) Increased or decreased of CTSA in glioma cancer compared to normal tissues in the GEPIA database. (C) Expression level of CTSA in glioma cancer was detected with UALCAN database. (D) Expression level of CTSA in glioma cancer tissues and normal tissues were determined with TCGA database.

### Associations between CTSA expression and clinicopathologic variables

**Figure 2:**
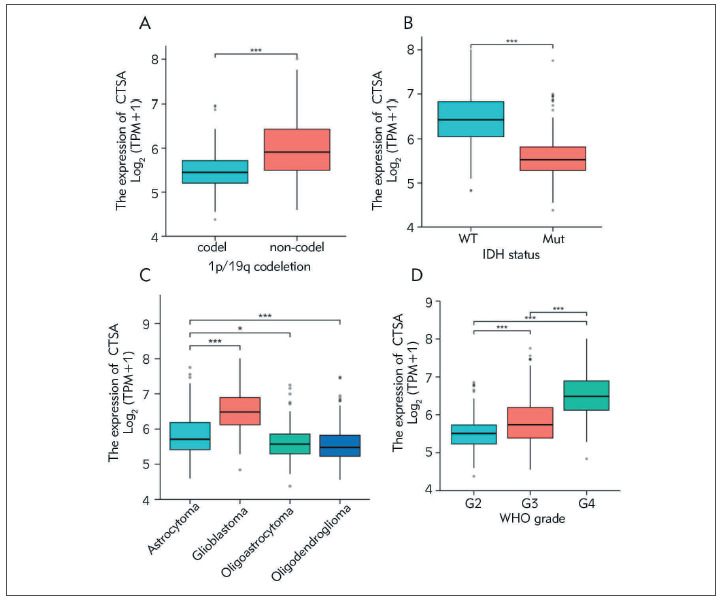
The correction of CTSA expression with clinicopathologic characteristics. (A) 1q/19q codeletion; (B) IDH status; (C) Histological type; (D) WHO grade.

Based on the analysis of giloma patients from the TCGA database, the Wilcoxon rank sum test showed that CTSA overexpression was significantly associated with 1p/19q codeletion and IDH (Figure 2A-Figure 2B). Multiple hypothesis testing (Dunn's test) using the Bonferroni method to correct for significance levels showed (Figure 2C) that G3 was higher than the mean of G2, with a statistically significant difference (P < 0.001); G4 was higher than the mean of G2, with a statistically significant difference (P < 0.001) The Kruskal-Wallis Test showed a statistically significant difference between the groups (P<0.001).

### Relationship between CTSA expression and prognosis of glioma

**Figure 3:**
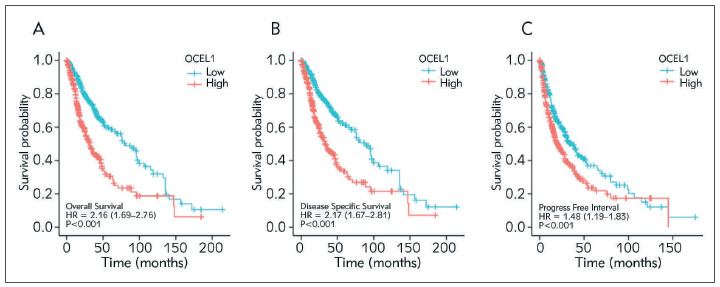
Kaplan-Meier survival curves comparing the high and low expression of CTSA in glioma. (A) Overall survival; (B) Disease Specific survival; (C) Progress free interval.

To assess the value of CTSA in predicting the prognosis of glioma patients, we analyzed the association of CTSA expression with OS, DSS and PFI. Results showed that the prognosis of glioma patients was worse in the high CTSA expression group (HR=2.16 (1.69–2.76) P<0.001) (Figure 3A). We further analyzed the DSS and PFI through the Kaplan-Meier Plotter database and found that the DSS (HR=2.17 (1.67–2.81) P<0.001) and PFI (HR=1.48 (1.19–1.83) P<0.001) were lower in glioma patients with high CTSA expression than in CTSA low expression group (Figure 3B-Figure 3C). These results suggested that patients with overexpressed CTSA had a worse prognosis.

### Identification of DEGs between the high and low CTSA expression groups

**Figure 4:**
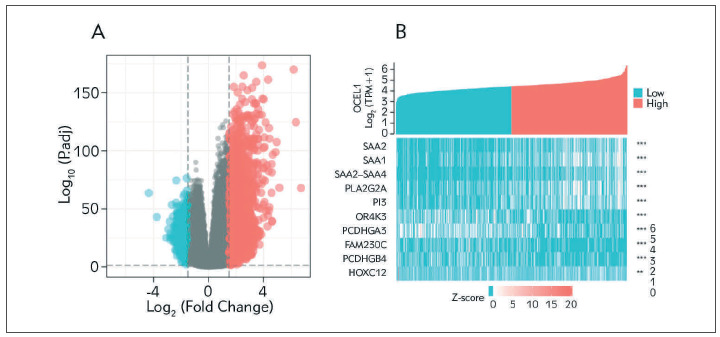
Differentially expressed genes between patients with high and low CTSA expression. (A) Volcanic map of differentially expressed genes between CTSA high expression group and low expression group. (B) Heat map of the first 10 significantly differentially expressed genes between CTSA high expression group and low expression group.

The data from TCGA was analyzed using the DSEeq2 package. The currently selected threshold was |log2(FC)|>1.5 & p<0.05, and the number of individuals meeting this threshold was 3337. Among, 2748 were defined as high expression (logFC>1.5) and 589 as low expression (logFC<-1.5) (Figure 4A). Figure 4B was a heat map of the top 5 CTSA high expression and the top 5 significantly differentially expressed genes in the low expression group, with red and green representing up- and down-regulated genes respectively.

### Correlation between immune cell infiltration and CTSA

**Figure 5:**
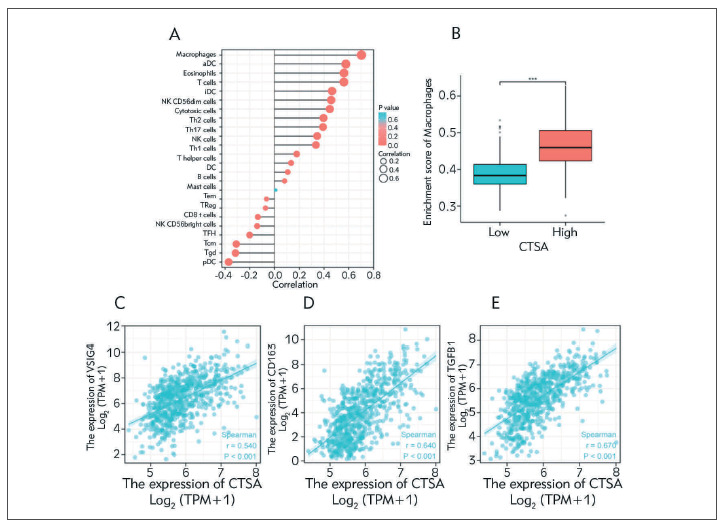
Association between immune cell infiltration and CTSA in glioma. (A) correlation between the relative abundance of 24 immune cells and CTSA expression level. (B) Macrophage infiltration level in the high CTSA expression group and low CTSA expression group in TCGA cohort. (C-E) correction between CTSA expression and M2-like macrophage marker.

We analyzed the correlation between the expression level of CTSA and the degree of immune cell enrichment according to Spearman's correlation coefficient pDC, Tcm and Tgd were negatively correlated with the expression level of CTSA and was positively correlated with the most abundance of macrophages (Figure 5A). More importantly, the overexpression of CTSA correlated with macrophages (Figure 5B). Therefore, we further analyzed the correlation between CTSA and the markers VSIG4, CD163 and TGFB1 in M2 macrophages (Figure 5C-Figure 5E). The results revealed that the expression levels of CTSA were negatively correlated with the markers VSIG4 (P<0.001, r=0.540), CD163 (P<0.001, r=0.640), TGFB1 (P<0.001, r=0.670) and significantly correlated with M2 macrophages.

## Discussion

Glioma, the most common malignant tumor of the central nervous system, with an increasing incidence rate in recent years [15]. Due to the high specificity and infiltration and growth of glioma cells, there is no obvious boundary between the tumor and normal brain tissue. Moreover, a larger number of micro invasive foci cannot be observed for now. Therefore, the operation can only remove the tumor on the premise of protecting brain function to the greatest extent and can't remove all the minimally invasive lesions. These residual micro invasive foci are the root cause of glioma recurrence in a short time. There are obvious molecular pathological changes before the change of tumor tissue morphology. Therefore, compared with histopathological classification, molecular pathological characteristics can more accurately guide the early diagnosis, treatment and clinical prognosis of glioma.

CTSA (cathepsin A), a lysosomal protease, protects b-galactosidase and neuraminidase-1 from proteolysis within the lysosome [16]. In addition to their intrinsic role in protein degradation, lysosomal proteins are also thought to play an important role in various types of tumors. The literature reports that the over expression of cathepsin can activate ErbB carcinogenic pathway [6], thereby promoting invasion and metastasis of breast cancer [7], pancreatic cancer [8], HCC [9] and colorectal cancer [10]. Since cathepsin D can mediate protease lectures and promote breast cancer invasion and metastasis, the concentration of cathepsin D in the cell membrane can be used to determine the metastasis of breast cancer [17]. In addition, inhibition of cathepsin K has been found to inhibit the progression of prostate cancer, while improving the therapeutic effects of zoledronic acid (ZA) [11]. Although many studies have found an effect of cathepsin on tumors, there is no reported correlation between CTSA and glioma.

In this study, bioinformatics analysis of the TIMER, UALCAN and TCGA public databases revealed higher levels of CTSA expression in glioma tissue than in normal glioma tissue. Overexpression of CTSA was significantly correlated with 1p/19q codeletion, IDH, WHO and histological types, suggesting that CTSA is undesirable clinicopathological factor. The clinical prognostic significance of CTSA in patients with glioma was then analyzed. In addition, Kaplan-Meier survival analysis showed that patients with CTSA overexpression in gliomas had significantly lower survival rates than those with low expression. Wang et al. [4] repotted that CTSA may serve as a potential diagnostic and prognostic. We therefore hypothesized that CTSA may also serve as a potential diagnostic and prognostic biomarker in glioma.

Tumor microenvironment immune cells are an important component of tumor tissue. Growing evidence demonstrated their clinicopathological significance in predicting the survival status and outcome of tumour patients [18] [19]. Specifically, the level of TAM infiltration regulates the progress of glioma [20]. TAM is mainly composed of M2 macrophages, and tumor immune infiltration analysis revealed that CTSA is closely related to immune-related pathways. By analyzing the relationship between CTSA and immune cell infiltration, we found that the level of M2 macrophage infiltration was significantly higher in the CTSA high expression group. In addition, the correlation between CTSA and immunosuppressive gene expression suggested that CTSA plays a key role in regulating tumor immunity.

In summary, the present study demonstrated that CTSA was overexpressed in glioma and could predict prognosis by integrating and analyzing glioma-related information from the TCGA database. It was also hypothesized that CTSA could be a potential target for glioma immunotherapy. It is suggested that CTSA may play a pro-carcinogenic role in the development of glioma and may be used as a biological marker to assess glioma risk classification, progress and targets for immunotherapy.

## Dodatak

### Conflict of interest statement

All the authors declare that they have no conflict of interest in this work.
